# Transcriptome profile changes in the jejunum of nonhuman primates exposed to supralethal dose of total- or partial-body radiation

**DOI:** 10.1186/s12864-023-09385-3

**Published:** 2023-05-22

**Authors:** Neetha Nanoth Vellichirammal, Sahil Sethi, Nagavardhini Avuthu, Stephen Y. Wise, Alana D. Carpenter, Oluseyi O. Fatanmi, Chittibabu Guda, Vijay K. Singh

**Affiliations:** 1 Department of Genetics, Cell Biology, and Anatomy, University of Nebraska Medical Center, Omaha, NE 68198 USA; 2 Division of Radioprotectants, Department of Pharmacology and Molecular Therapeutics, F. Edward Hébert School of Medicine, Uniformed Services University of the Health Sciences, Bethesda, MD USA; 3 Armed Forces Radiobiology Research Institute, Uniformed Services University of the Health Sciences, Bethesda, MD USA

**Keywords:** Gamma tocotrienol, Jejunum, Nonhuman primates, Radiation injury, Radiation countermeasure, Transcriptomics, Total-body irradiation, Partial-body irradiation

## Abstract

The risk of exposure of the general public or military personnel to high levels of ionizing radiation from nuclear weapons or radiological accidents is a dire national security matter. The development of advanced molecular biodosimetry methods, those that measure biological response, such as transcriptomics, to screen large populations of radiation-exposed victims is key to improving survival outcomes during radiological mass casualty scenarios. In this study, nonhuman primates were exposed to either 12.0 Gy cobalt-60 gamma (total-body irradiation, TBI) or X-ray (partial-body irradiation, PBI) 24 h after administration of a potential radiation medical countermeasure, gamma-tocotrienol (GT3). Changes in the jejunal transcriptomic profiles in GT3-treated and irradiated animals were compared to healthy controls to assess the extent of radiation damage. No major effect of GT3 on radiation-induced transcriptome at this radiation dose was identified. About 80% of the pathways with a known activation or repression state were commonly observed between both exposures. Several common pathways activated due to irradiation include FAK signaling, CREB signaling in the neurons, phagosome formation, and G-protein coupled signaling pathway. Sex-specific differences associated with excessive mortality among irradiated females were identified in this study, including Estrogen receptor signaling. Differential pathway activation was also identified across PBI and TBI, pointing towards altered molecular response for different degrees of bone marrow sparing and radiation doses. This study provides insight into radiation-induced changes in jejunal transcriptional profiles, supporting the investigation for the identification of biomarkers for radiation injury and countermeasure efficacy.

**Supplementary Information:**

The online version contains supplementary material available at 10.1186/s12864-023-09385-3.

## Introduction

Acute radiation syndrome (ARS) is an illness in humans involving multiple organ systems caused by total- or partial-body exposure to radiation of greater than 2 Gy at a high dose rate. There are three sub-syndromes of ARS characterized both by the different organ systems affected and by the aggregate absorbed dose of radiation. These include the hematopoietic (H-ARS; 2 – 6 Gy), gastrointestinal (GI-ARS; 6 –10 Gy), and neurovascular sub-syndromes (NV-ARS; > 10 Gy) [1–3]. Individuals developing H-ARS or GI-ARS are considered to benefit from treatment with radiation medical countermeasures (MCMs) and have generally been the focus of research efforts for developing MCMs and for the identification of biomarkers. Individuals exposed to supralethal doses of radiation developing NV-ARS, on the other hand, are considered incurable with MCMs. In case of such supralethal doses of radiation exposure leading to NV-ARS, death occurs within 24 to 48 h after exposure [4].

The development of MCMs is a top priority for the government due to the increased risk of the dispersion of radioactive materials by domestic or foreign adversaries, or from a nuclear event or radiological accidents. All four radiation MCMs currently approved by the US Food and Drug Administration (US FDA) are radiomitigators for H-ARS, which are for use after radiation exposure but before the onset of symptoms [5, 6]. No radioprotector has been approved by the US FDA. One potential radiation MCM under advanced development as a pre-exposure prophylaxis for H-ARS is gamma-tocotrienol (GT3), a component of vitamin E [7]. GT3 is an antioxidant as well as an inhibitor of 3-hydroxy-3-methylglutaryl-coenzyme A reductase, and has proven radioprotective efficacy in both mice and nonhuman primates (NHPs) when administered 24 h prior to total-body irradiation (TBI) [8–12]. Its dose reduction factor in mice is 1.29 when administered 24 h prior to irradiation at a dose of 200 mg/kg [13]. This agent is being developed as a radiation MCM for H-ARS following the US FDA Animal Rule since the efficacy of such agents cannot be investigated in a clinical setting under phase II and phase III due to ethical reasons [14]. The NHP model is considered the gold standard of animal models for developing radiation MCMs, and identifying and validating biomarkers for radiation injury and countermeasure efficacy [15]. NHPs are as close to humans as possible due to 95% DNA homology as well as similar pathophysiology and organ structure.

Clinical interventions to treat ARS include the use of MCMs and other treatment options depending on the absorbed radiation dose. Therefore, it is critical to assess the extent of radiation exposure in order to provide appropriate medical interventions [16]. However, current dosimetry methods used to assess the absorbed radiation dose, including cytogenetic analysis of peripheral blood lymphocyte assays, are time-consuming, labor-intensive, and require well-trained personnel. In a radiological/nuclear disaster scenario, the infrastructure and personnel required to perform these arduous assays would be scarce [17]. Therefore, new high-throughput screening tools that are fast and easy to use are needed to screen subjects at a population scale quickly and efficiently so medical interventions can begin soon after exposure to improve survival outcomes after a mass casualty scenario. Some promising methods include assays involving genomics, transcriptomics, proteomics, and metabolomics, which have the potential for high-throughput mass screening and can reliably account for population variability [18–20]. Due to technological advancements to accurately sequence transcripts and the availability of bioinformatics algorithms to analyze these profiles, transcriptomic profiling is favored as a high throughput, reliable, and accurate approach for assessing radiation-induced damage and for identifying radiation-specific biomarkers [21–24].

In this study, we profiled transcriptomic changes in the jejuna, a very sensitive and important tissue to assess radiation injury, of NHPs (rhesus macaques, *Macaca mulatta*) exposed to a supralethal dose of radiation-inducing ARS. We also compared the gene expression changes resulting from TBI and partial-body irradiation (PBI). Furthermore, the changes induced by the promising MCM for H-ARS, GT3, were also analyzed. The sex-dependent transcriptional changes in response to GT3 and irradiation were evaluated.

## Results

### Jejunum transcriptome profiling for radiation response and drug response

For each experimental group, the effect of irradiation (healthy vs. irradiated), sex (male vs. female), drug treatment (GT3 vs. vehicle), and days post-exposure (d 7 vs. d 4) were summarized using RNA-seq analysis. A list of evaluations examined in this study with the differentially expressed genes is presented in Table 1.

**Table 1:** Different conditions analyzed in this study are listed in this table

Radiation	Comparison	Up	Down	Total DE
**PBI**	Healthy vs PBI-Vehicle	2,350	1,364	3,714
	Healthy vs PBI-GT3	2,992	1,704	4,696
	PBI-M-GT3 vs PBI-F-GT3	21	13	34
	PBI-M-vehicle vs TBI-F-vehicle	21	13	34
	PBI-vehicle-SD4 Vs PBI-GT3-SD4	16	21	37
	PBI-vehicle-SD7 Vs PBI-GT3-SD7	27	5	32
	PBI-vehicle Vs TBI-GT3 (all)	1	1	2
**TBI**	Healthy vs TBI-Vehicle	2,644	1,799	4,443
	Healthy vs TBI-GT3	2,692	1,804	4,496
	TBI-vehicle vs TBI-GT3	0	0	0
	TBI-vehicle-SD4 vs TBI-GT3 -SD4	5	9	14
	TBI-vehicle-SD7 vs TBI-GT3-SD7	4	8	12
	TBI-M-GT3 vs TBI-F-GT3	53	53	106
	TBI-M-vehicle vs TBI-F-vehicle	5	8	13
**TBI/PBI**	TBI-M-GT3 PBI-M-GT3	52	58	110
	TBI-GT3 Vs PBI-GT3	150	274	424
	TBI-vehicle vs PBI-vehicle	51	51	102
	TBI-F- GT3 Vs PBI-F- GT3	112	90	202

Differentially regulated genes from RNA-seq data using *DESeq2* analysis are shown here

*PBI* Partial-body irradiation, *TBI* Total-body irradiation, *M* Male, *F* Female, *SD4* Study day 4, *SD7* Study day 7, *DE* Differentially expressed genes

### Transcriptomic profile comparisons to understand the effect of TBI

**Fig. 1:**
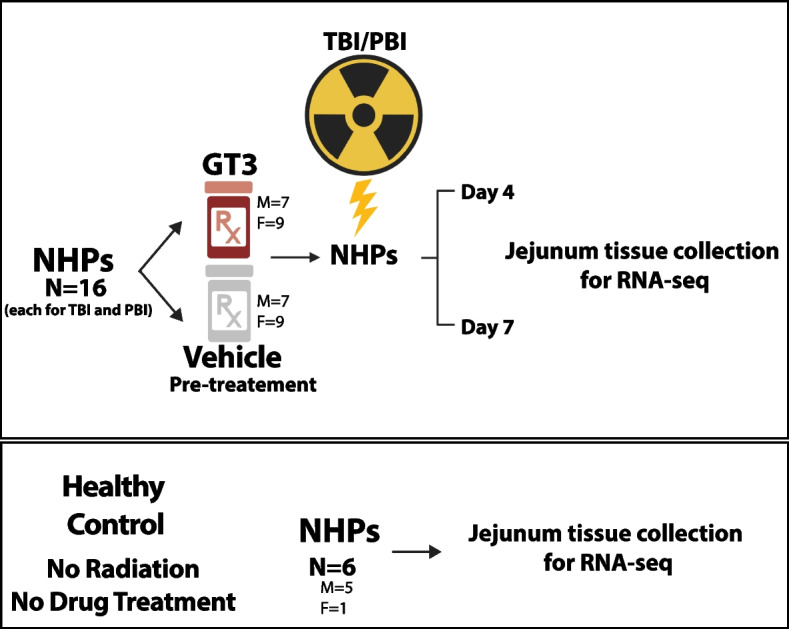
Experimental design. Nonhuman primates were exposed to either total-body radiation (TBI) or partial-body radiation (PBI). They were compared to Controls to identify differentially regulated genes associated with radiation exposure. TBI or PBI NHPs were also treated with gamma-tocotrienol (GT3) or vehicle 24 h before radiation. Jejunum tissue for RNA-seq analysis was collected at Day 4 and Day 7 post-exposure

Changes in the jejunum transcriptome due to 12 Gy ^60^Co γ-radiation were profiled by comparing the irradiated NHPs administered vehicle (designated as TBI-Veh) and untreated/unirradiated healthy animals (designated as Control) (Fig. 1).

**Fig. 2:**
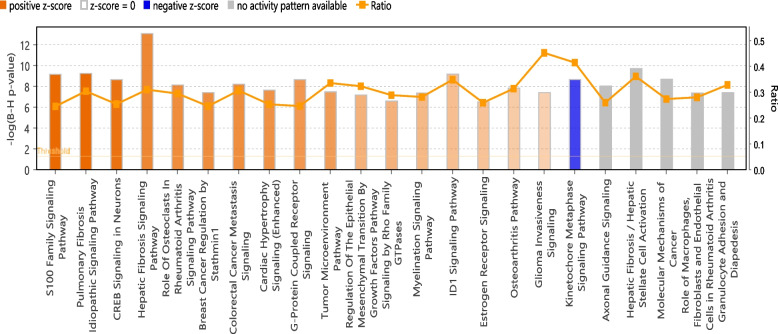
Enriched canonical pathways identified using *IPA* in TBI treatment. Both up and downregulated genes in each comparison are represented here (Benjamini–Hochberg corrected *p*-value ≤ 0.05). Orange or shades of orange bars indicate the predicted activation state of the canonical pathway, and blue or lighter shades of the blue bar indicate a negative z-score and down-regulation of the pathway. Grey bars represent pathways with no predicted activity pattern. The ratio indicates the number of significantly enriched genes compared with the total number of genes associated with that canonical pathway

A total of 4,443 genes were differentially expressed after irradiation (2,644 and 1,799 genes upregulated or downregulated in TBI-vehicle, respectively, Supplementary Table 1). Ingenhuity pathway analysis (IPA) of these differentially expressed genes revealed a large number of upregulated pathways associated with FAK signaling, CREB signaling, Wound healing signaling, p38 MAPK signaling, Phagosome formation , and G-Protein coupled signaling. On the other hand, pathways associated with PTEN signaling, Kinetochore metaphase signaling pathway and PPAR Signaling were downregulated in TBI-Veh (Fig. 2, Supplementary Table 2).

**Fig. 3:**
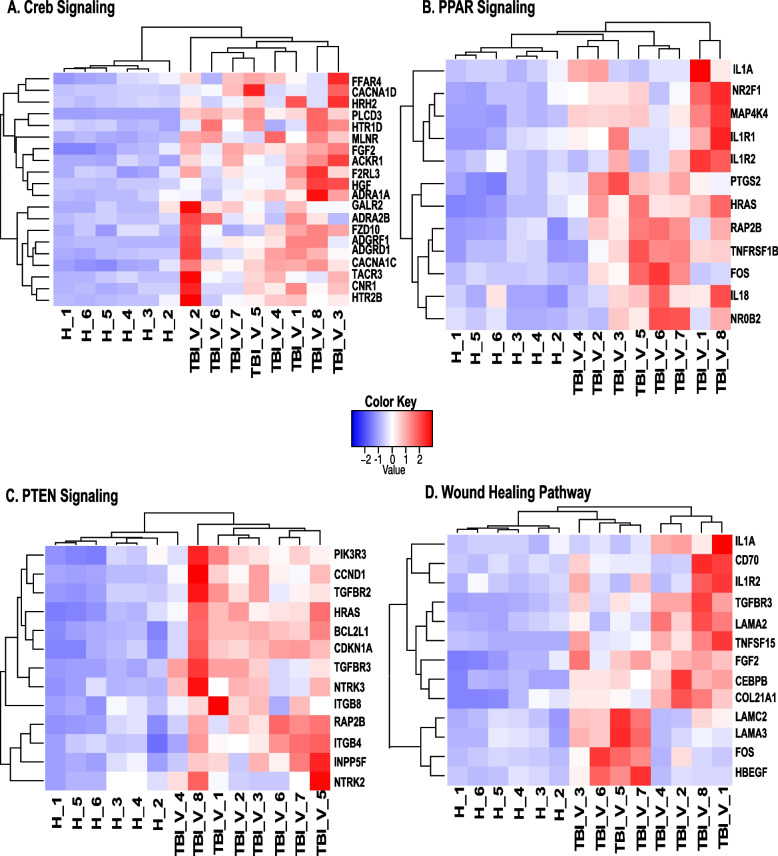
Heatmap of genes in the differentially regulated pathways associated with TBI in jejunum. **A**. CREB Signaling pathway. **B**. PPAR Signaling pathway. **C**. PTEN Signaling Pathway. **D**.Wound Healing Signaling Pathway. Row wise clustering indicates genes clustered according to their expression. Column wise clustering separates experimental groups. Red indicates upregulation and blue indicates downregulation

Differentially expressed genes in some of the enriched pathways are represented in Fig. 3.

**Fig. 4:**
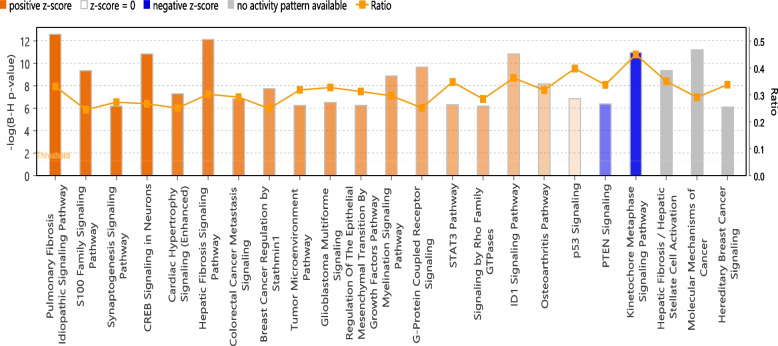
Enriched canonical pathways identified using IPA in TBI compared to GT3 pretreatment. Canonical pathways enriched in Control vs. TBI-GT3. Both up and downregulated genes in each comparison are represented here (Benjamini–Hochberg corrected *p*-value ≤ 0.05). Orange or shades of orange bars indicate the predicted activation state of the canonical pathway, and blue or lighter shades of the blue bar indicate a negative z-score and down-regulation of the pathway. The activity patterns are not predicted in the pathways with gray bars. The ratio indicates the number of significantly enriched genes compared with the total number of genes associated with that canonical pathway

We also compared the transcriptomic changes in the jejunum of NHPs administered GT3 prior to TBI (TBI-GT3) to control. A total of 4,496 genes were differentially expressed in this comparison, with 2,692 genes upregulated in TBI-GT3 (Supplementary Table 3). IPA revealed several pathways up or downregulated, similar to TBI-Veh vs. Control (Fig. 4, Supplementary Table 4).

Several pathways were upregulated, including CREB signaling, Phagosome formation, STAT3 pathway, and GP6 signaling. PTEN signaling, Cell cycle control of chromosomal replication, Oxidative phosphorylation, PPAR Signaling, and Kinetochore metaphase signaling pathways were downregulated in TBI-Veh. A comparison of pathways differentially regulated in TBI-GT3 vs. Control and TBI-Veh vs. Control were performed using IPA (Supplementary Fig. 1). Several pathways, including Cell cycle control of chromosomal replication, PTEN signaling and Kinetochore metaphase signaling pathways, were exclusively downregulated in TBI-GT3 vs Control, while RHOGDI signaling downregulation was exclusive in TBI-Veh vs. Control. On the other hand, several pathways including Phagosome formation, G-protein coupled receptor signaling, Role of osteoclasts in Rheumatoid Arthritis signaling pathway and GP6 signaling pathways were upregulated in Control vs. TBI-Veh.

The effect of TBI on sex was also compared in this study. A total of 106 genes (53 up and 53 downregulated in TBI-GT3) were differentially expressed in male NHPs pre-treated with GT3 and irradiated compared to corresponding females (Supplementary Table 5). No enriched IPA pathways after false discovery rate (FDR) correction were identified in this comparison. Very few genes were differentially expressed in jejunum when male and female NHPs were exposed to radiation and treated with vehicle (TBI-Veh) were compared (N = 13, five genes upregulated and eight genes downregulated in males, Supplementary Table 6).

Transcriptional profiles of TBI NHPs pre-treated with GT3 or vehicle were also compared to understand the molecular pathways altered by drug exposure duration. We did not find any genes differentially regulated when the transcriptional changes on days 4 and 7 were compared together (TBI-GT3 vs. TBI Veh). However, a handful of genes were differentially regulated when transcriptional changes between TBI-GT3 and TBI-Veh on day 4 (TBI-GT3-SD4 vs. TBI Veh) and day 7 (TBI-GT3-SD7 vs. TBI-Veh) were compared (Supplementary Tables 7 and 8).

### Transcriptomic profiling to understand the effects of PBI and GT3 pretreatment to NHP on the jejunum tissue

**Fig. 5:**
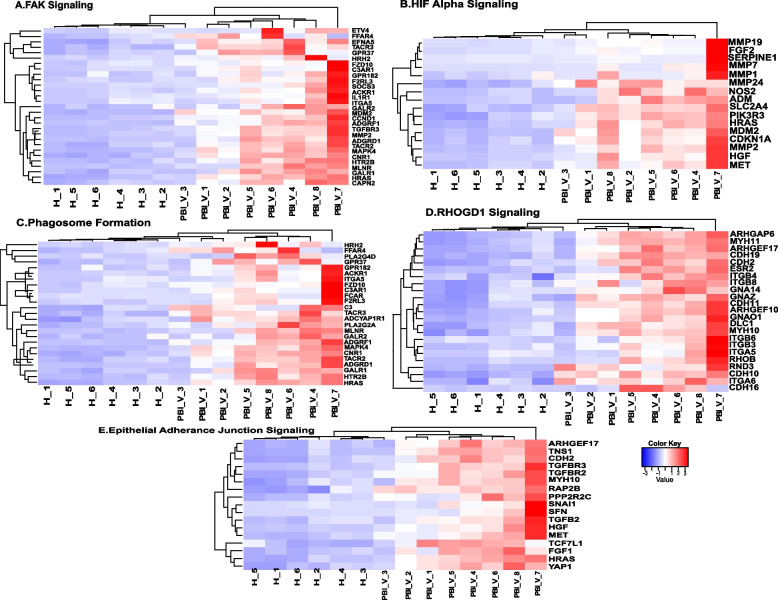
Heatmap of genes in the differentially regulated pathways associated with PBI in jejunum. **A.** FAK Signaling pathway. **B.** HIF Alpha Signaling pathway. **C.** Phagosome formation. **D.** RHOGDI Signaling Pathway. **E.** Epithelial Junction Signaling. Row wise clustering indicates genes clustered according to their expression. Column wise clustering separates experimental groups. Red indicates upregulation and blue indicates downregulation

Transcriptomic changes in the jejunum due to PBI with 12 Gy LINAC-derived X-ray were profiled by comparing the vehicle-treated irradiated NHPs (designated as PBI-Veh) and unirradiated Control (designated as Control). A total of 3,714 genes were differentially expressed after irradiation (2,350 and 1,364 genes upregulated and downregulated in PBI-Veh, respectively, Supplementary Table 9). IPA of these differentially expressed genes revealed activation of several pathways associated with phagosome formation, CREB signaling in neurons, HIF1α signaling, and GP6 signaling pathway after PBI (Supplementary Fig. 2, Supplementary Table 10). Genes differentially expressed in FAK signaling, HIF Alpha signaling, Phagosome formation, RHOGDI signaling, and Epithelial adherence signaling are represented in Fig. 5.

On the other hand, PTEN signaling, RHOGDI signaling, Oxidative phosphorylation, and Epithelial adherence junction signaling were downregulated after PBI. Several common pathways associated with transcriptional changes were identified in PBI and TBI, including FAK signaling, Phagosome formation, G-Protein Coupled Receptor Signaling, and IL-6 Signaling.

Similarly, differential expression analysis of NHPs of PBI and pre-treated with GT3 (PBI-GT3) compared to Control identified 4,696 genes (2,992 and 1,704 genes with statistically different gene expression, Supplementary Table 11). IPA revealed pathways associated with FAK signaling and phagosome formation. CREB signaling in neurons and G-Protein coupled signaling were activated after PBI-GT3 exposure (Supplementary Fig. 3, Supplementary Table 12). Pathways associated with PTEN signaling, RHOGDI signaling, LXR/RXR activation, and PPAR signaling were downregulated. We also identified several common altered pathways resulting from PBI-Veh vs. Control and PBI-GT3 vs. Control, such as activation of pathways associated with FAK signaling, Phagosome formation, and wound healing pathway after PBI (Supplementary Fig. 4). On the other hand, pathways associated with PPAR Signaling, RHOGDI Signaling, and PTEN Signaling were downregulated in both comparisons.

Sex-specific transcriptional differences associated with PBI were also studied. Sex differences among NHPs administered either vehicle or GT3 with PBI had very few transcriptional changes reflected in the jejunum (comparison: PBI-M-Veh vs. PBI-F-Veh and PBI-M-GT3 vs. PBI-F-GT3, Supplementary Table 13A and B). In addition, the differentially expressed genes also overlapped to a large extent (59% of genes had a similar transcriptional response). IPA identified pathways associated with HIF1α signaling, wound healing signaling and HOTAIR regulatory pathway in male and female NHPs administered the vehicle (Supplementary Fig. 5, Supplementary Table 14). We did not find any FDR corrected differentially regulated pathways among the male and female NHPs pre-treated with GT3.

The differences in the transcriptional response to GT3 after irradiation on day 4 and day 7 were also studied. The number of genes differentially expressed at both days post-irradiation was similar (comparisons: PBI-Veh-SD4 vs. PBI-GT3-SD4 and PBI-Veh-SD7 vs. PBI-GT3-SD7 Supplementary Table 15A, B). No differentially regulated pathways were identified on day 7 post-exposure. When both samples collected on day 4 and day 7 post-exposure were pooled and analyzed, only two genes were found to be differentially expressed (comparison: PBI-Veh vs. PBI-GT3).

### Comparison of transcriptional changes associated with TBI and PBI in the jejunum

Transcriptional changes in jejunum associated with TBI and PBI could be different. To understand these transcriptional changes, we compared the profile of TBI and PBI in the jejunum. The extent of transcriptional differences across TBI and PBI after pre-treatment with GT3 (TBI-GT3/PBI-GT3) were larger than just the vehicle-treatment (TBI-Veh/PBI-Veh). A total of 424 genes were differentially expressed in the TBI-GT3 vs. PBI-GT3 comparison (150 genes upregulated and 274 genes downregulated in TBI-GT3, Supplementary Table 16). No pathways were differentially regulated in this comparison. Comparison of NHPs pre-treated with the vehicle and either TBI or PBI identified 102 differentially regulated (51 genes up or down-regulated, Supplementary Table 17). No differentially regulated IPA pathways were identified in this comparison.

Sex-specific comparisons across TBI and PBI were also performed. Among GT3-treated TBI and PBI, females had higher differentially expressed genes in the jejunum than males (Supplementary Tables 18A and B).

IPA match analysis of NHPs pre-treated with the vehicle and TBI or PBI compared to healthy unexposed animals identified several common pathways. These include Pulmonary fibrosis idiopathic signaling pathway, S100 family signaling pathway, Synaptogenesis signaling pathway, CREB signaling in neurons, and Tumor microenvironment pathway among others that were activated in both exposures (Supplemental Fig. 6). On the other hand, Oxidative phosphorylation, Kinetochore metaphase signaling pathway, PTEN signaling, and RHOGDI signaling pathways were commonly downregulated.

## Discussion

The purpose of this study was to identify biomarkers for radiation injury as well as for the development of MCMs against GI-ARS. While reports have shown that survival prospects are higher for H-ARS after irradiation [25–27], the focus is now shifting towards GI-ARS. However, the development of effective MCMs against GI-ARS is limited due to insufficient data associated with this sub-syndrome. To address this issue, we have profiled the transcriptomic changes in the jejunum associated with TBI or PBI. We selected jejunum as the focus of this study, because this is an important organ for radiation injury and an active part of the GI system that secretes enzymes.

Biomarkers play a vital role in radiation dose exposure assessment as well as for the development of MCMs. The US FDA Animal Rule is being used to develop MCMs where clinical Phase II and III efficacy studies are not feasible due to ethical reasons and hence, drugs receive regulatory approval using NHP efficacy data. Therefore, biomarkers are necessary for drug dose conversion from animal models to humans, which is a crucial step in MCM development.

A comparison of transcriptional changes in the jejunum due to TBI-Veh vs. Control and PBI-Veh vs. Control identified common and unique pathways associated with TBI and PBI. Approximately 80% of the pathways with known activation or repression state (non-zero Z-score from IPA) were shared between TBI and PBI when compared to their respective Controls, indicating similar response patterns. Common pathways activated by radiation injury included the FAK signaling, CREB signaling in neurons, phagosome formation, and G-protein coupled signaling pathways (Supplementary Fig. 6). On the other hand, PPAR signaling, RHOGDI signaling, PTEN signaling, Hippo signaling, and Epithelial adherens junction signaling were downregulated after irradiation. In previous studies, it was found that changes in epithelial junctions in oral and colon cells were linked to side effects caused by irradiation [28–30]. Intestinal epithelial junction proteins play a critical role in maintaining the physical barrier and regulating the movement of ions, solutes, and water across the epithelium [31]. During later stages of exposure, an increase in the expression of adherens junctions was noted, indicating that the epithelium is reorganizing and proliferating, which may lead to loss of radiosensitivity [32].

The Hippo pathway is involved in the regulation of normal intestine homeostasis. It is inhibited under conditions that promote growth, stabilizing YAP1 and TAZ coactivators and activating the cascade for repressing apoptosis and triggering cell proliferation [33]. YAP1 is crucial for intestinal regeneration and recovery after injury. It was also found to be activated a few days after irradiation in mice intestinal epithelial cells [34]. Our study also found YAP1 in the Hippo pathway upregulated in the jejunum after both TBI and PBI. Additionally, we observed that Wnt signaling was also activated by YAP1 after irradiation.

Several pathways unique to TBI or PBI were also identified; for example, EGF signaling was activated with PBI but not with TBI. It has been reported that radiation exposure induces EGFR expression [35], and cytotoxicity is inversely correlated to EGFR expression [36–38]. Since this pathway was not activated after TBI with gamma-ray, it can be speculated that EGFR expression is associated with X-ray PBI and not with TBI γ-radiation. Interestingly, the pyroptosis signaling pathway was activated in TBI but not in PBI. Pyroptosis is an inflammatory cell death associated with microbial infection and is complemented by the activation of inflammasomes. Pyroptosis has been associated with radiation therapy in bone marrow-derived macrophages [39]. Reports also suggest a direct association with γ-radiation-induced pyroptosis at a dose higher than 7.5 Gy in microvascular endothelial cells, indicating that ionizing radiation can stimulate inflammasome-mediated cell death [40]. We also need to consider the differences in the radiation source (gamma vs. X-ray) when comparing transcriptional changes across TBI and PBI, as the biological effects of radiation depend on the radiation energy and a comparison of radiation sources needs assessment of energy and filtration of the beam [41]. Orthovoltage X-rays were reported to produce severe hematopoietic, immunologic, and GI injury compared to metavoltage γ-rays at a constant dose. Though we have used X-ray for PBI, our X-ray source is LINAC (not orthovoltage).

The transcriptional effect of TBI on jejunum and lungs [42] was drastically different, with the jejunum showing larger changes than the lungs. Only a few common pathways were upregulated after TBI in both tissues including PD-1, PD-L1 cancer immunotherapy pathway, Xenobiotic metabolism PXR signaling pathway, GADD45 signaling, and p53 signaling (Supplementary Table 19, Supplementary Fig. 7A, B). However, several pathways were downregulated after TBI in both tissues, including the Neutrophil extracellular Trap signaling pathway, Kinetochore metaphase signaling pathway, Cyclins and cell cycle regulation, and Cell cycle control of chromosomal replication. In the jejunum, there was inhibition of the role of BRCA1 in DNA damage response, Epithelial adherens junction signaling, PTEN signaling, RHOGDI signaling, and EIF2 signaling. On the other hand, these pathways were activated in the lung after TBI. Furthermore, many pathways were strongly inhibited in the lungs after TBI, such as the Pathogen induced cytokine storm signaling pathway, cardiac hypertrophy signaling by stathmin1, FAK signaling, CREB signaling in neurons, S100 family signaling pathway and Phagosome pathways, which were all activated in the jejunum.

Transcriptional change induced by PBI on jejunum and lungs [42] was also compared (Supplementary Fig. 8A, B). Our study found that PBI induced activation of several pathways in the jejunum compared to the lungs, while a number of pathways were downregulated in the lung after PBI. Some of the pathways that were upregulated in the jejunum and downregulated in the lungs after PBI included FAK signaling, Phagosome formation, Tumor microenvironment pathway, Pathogen induced cytokine storm signaling pathway, G-protein coupled receptor signaling, CREB signaling in neurons, and S100 family signaling pathway. These findings indicate that the effect of PBI varies across different tissues. PBI also caused activation of different pathways in the jejunum and lungs. Additionally, p53 signaling and PD-1, and the PD-L1 cancer immunotherapy pathway were upregulated in both jejunum and lungs after PBI. However, Neutrophil extracellular Trap signaling pathway, and Crosstalk between dendritic cells and natural killer cells were downregulated in both tissues (Supplementary Fig. 8B).

Comparison of transcriptional changes in jejunum associated with GT3 treatment in both TBI and PBI was limited, indicating that GT3 was minimally effective in counteracting radiation exposure at such a supralethal dose. This observation was consistent with the effect of GT3 in irradiated lung tissue, implying that GT3-induced transcriptional changes were minimal in different tissues. Our recent study on the conventional GI parameters used in radiobiology to assess MCM efficacy (crypt survival, crypt depth, crypt cell proliferation, apoptotic cell death, villus height, mucosal surface area, and tight junction related proteins) also demonstrated limited efficacy of GT3 at 12 Gy TBI [42]. However, analysis of the impact of GT3 treatment on day 4 and day 7 revealed a handful of differentially expressed genes, with PBI having more genes differentially expressed than TBI, and the effect being more pronounced on day 4. This suggests that GT3 interaction was slightly more distinct in PBI than in TBI and decreased with time. Of note, *NKAIN4* (Sodium/Potassium Transporting ATPase Interacting 4), a gene known to increase ionizing radiation sensitivity, was downregulated in GT3-treated jejunum but upregulated in jejunum after TBI. GT3 treatment led to a decrease in *NKAIN4* expression in the jejunum, indicating that the drug can counter radiation injury.

In our study, we found that radiation-induced changes differed between male and female NHPs, which is consistent with a previous study on rhesus macaques that reported higher mortality among females after radiation exposure [43]. This highlights the importance of considering sex-specific differences when developing MCMs. In particular, we observed larger sex-specific differences in the jejunum after PBI compared to TBI. Our recent study also showed that female NHPs are more sensitive to radiation exposure than males [44]. Inhibition of Matrix metalloproteases, Estrogen receptor signaling, HIF1α signaling, EIF2 signaling, and HOTAIR regulatory pathways were differentially regulated across males and females with PBI (Supplementary Table 14). Further investigation of these pathways is necessary to fully understand the sex-specific radiation induced-changes in the jejunum.

## Conclusions

Our study profiles the transcriptomic changes in the jejunum associated with TBI and PBI. We identified that the radiation MCM, GT3, induced minimal changes in the transcriptional profile of jejunum after supralethal TBI or PBI with 12 Gy to NHPs. Since there is no conclusive data to understand the role of GT3 as a radioprotectant at such a supralethal dose of radiation, further study is needed to understand the role of GT3 as a MCM for GI-ARS. Sex-specific differences that might be linked to higher mortality among irradiated females were identified in this study, including Estrogen receptor signaling. Differential pathway activation was also identified across PBI and TBI which points towards molecular response associated with different degrees of bone marrow sparing and radiation doses. The unique pathways associated with PBI or TBI in the jejunum could be investigated further for characterizing biomarkers for radiation exposure.

## Material and methods

### Animals and experimental design

A total of 32 naïve rhesus macaques (*Macaca mulatta*, Chinese sub-strain, 14 males and 18 females) were used for this study. They were between 3.5 – 5.5 years of age, weighing 4.35 – 10.35 kg. Each group of 16 animals for TBI and PBI was randomly divided; eight received GT3 (37.5 mg/kg, subcutaneously (*sc*)), and the remaining eight received vehicle. All animals were maintained in a facility accredited by the Association for Assessment and Accreditation of Laboratory Animal Care (AAALAC)-International. Animals were quarantined for six weeks prior to the initiation of the experiment. Animal housing, health monitoring, care, and enrichment during the experimental period have been described in detail earlier [45, 46]. Details of the sex of the animals used in each group is provided in Supplementary Table 20. All animal procedures and methods were performed in accordance with the relevant guidelines and regulations. All animal studies were approved by the Institutional Animal Care and Use Committee (BIOQUAL Inc., protocol #18–060) and the Department of Defense Animal Care and Use Review Office (ACURO). This study was carried out in strict accordance with the recommendations made in the *Guide for the Care and Use of Laboratory Animals* [47].

### Drug preparation and administration

An olive oil formulation was used as the vehicle control. GT3 and vehicle formulations (50 mg/ml) in 5% Tween-80 in saline were purchased from Callion Pharma (Jonesborough, TN, USA). The quantity of GT3 or vehicle for each animal was determined based on NHP body weight. The dose of GT3 administered was 37.5 mg/kg *sc* 24 h prior to irradiation, as described earlier [11].

### Radiation exposure

For TBI and PBI, the high level ^60^Co gamma irradiator and linear accelerator (LINAC) were used, respectively.

#### Total-body irradiation

NHPs were fasted for approximately 12 h prior to irradiation, transported, sedated, and exposed to a midline dose of 12 Gy ^60^Co γ-radiation at a dose rate of 0.6 Gy/min (bilateral, simultaneous exposure), as described earlier [48, 49]. This group contained 3 males and 5 females each in the GT3 treated group as well vehicle controls.

#### Partial-body irradiation

NHPs were fasted, transported, and sedated as described above for TBI. For PBI, NHPs were irradiated one at a time using a 4 MV photon beam from an Elekta Infinity clinical LINAC. Anterior/posterior (AP) measurements of the NHPs at the location of the absorbed dose target (core of the abdomen) were measured with a digital caliper. Irradiation procedures for exposure with LINAC and dosimetry are described earlier [42]. We had to use two different radiation sources for PBI and TBI due to specific reasons. LINAC is preferred for PBI as it provides a collimated beam and the irradiation field is limited. Therefore, we can place only the part of the body under the field one is interested to expose. The high level cobalt facility for gamma-irradiation has a panoramic field and it is not possible to provide complete sparing of any body part one is interested to spare. This group contained 4 males and 4 females each in the GT3- treated as well vehicle.

### Euthanasia

Animals exposed to 12 Gy, either total-body or partial-body radiation, were euthanized either on day 4 or day 7 post-irradiation. Euthanasia was carried out per the American Veterinary Medical Association (AVMA) guidelines using EUTHASOL Euthanasia Solution (pentobarbital sodium and phenytoin sodium) when animals reached a point of no return [50].

### Jejunum tissue collection

As soon as the jejuna samples were harvested, all samples were transferred into individual sterile storage tubes and placed on dry ice, which was then stored at -80 °C until use. Six animals (3 vehicle/3 GT3) were euthanized as scheduled on day 4 post-irradiation for the TBI study. The remaining ten animals (5 vehicle/5 GT3) were euthanized on day 7, and tissue samples were collected accordingly. Similarly, for the PBI study, tissue samples from six animals (3 vehicle/3 GT3) were collected according to schedule on day 4, while all remaining samples (5 vehicle/5 GT3) were collected on day 7 post-irradiation. In addition, tissue samples harvested from healthy/unirradiated animals were used as the control. The experimental design is presented in Fig. 1.

### RNA isolation, library preparation and sequencing

Total RNA was isolated from frozen jejunum tissue as per the manufacturer's protocol for the RNeasy Lipid Tissue Mini kit (Qiagen, Germantown, MD, USA) and was quantified using Qubit 4 fluorometer (Invitrogen, Carlsbad, CA). Quality and quantity of RNA was determined as previously published, and Illumina libraries prepared as per manufacturer instructions [42]. Briefly, 500 ng of total RNA was inputted using the TrueSeq stranded mRNA Library Prep kit (Illumina, San Diego, CA) and sequencing were performed on the NextSeq 500 (Illumina) with paired-end reads of 75 base pairs in length.

### Data processing and analysis

Sequencing data were demultiplexed, and FASTQ files were generated using *bcl2fastq2* software (Illumina, version 2.20.0). Sequencing quality control was performed using *FastQC* tool [51]. The reads were aligned to the macaque genome *Macaca mulatta* Mmul_10.105 using the *STAR* spliced read aligner in the two-pass mode [52] and the latest Ensembl gene transfer format (GTF) file. The average percent alignment percentage of uniquely mapped reads ranged from 86 to 92, with an average of 90% across samples. Filtering of genes based on median gene expression profile above 10 RNA-seq read counts was performed. Low expressed genes were filtered out and 47% of the total genes analyzed remained in the downstream analysis.

Read counts for each gene were calculated using *STAR* aligner and differentially expressed genes across different comparisons were identified using *DESeq2* [53] as described earlier [42]. A gene was identified as differentially expressed if the FDR cutoff using the Benjamini–Hochberg procedure for multiple testing correction was ≤ 0.05 and the absolute fold change was above 1.5. TBI and PBI assessments were performed as separate conditions. *DESeq2* multi-factor design was used to analyze the paired samples in this study, including irradiation, drug treatment, sex, and days post-irradiation.

Differentially expressed genes in different comparisons were used to perform pathway analysis using IPA (QIAGEN Inc.) and KEGG pathways using the *ShinyGO* v0.741 [54, 55]. In all comparisons, *KEGG* pathways of the up- and down-regulated genes were performed separately to reveal the differentially regulated pathways. A comparison of enriched pathways across different conditions was performed using *IPA* match analysis. All abbrevations used are provided in Supplementary Table 21.

## Supplementary Information

**Additional file 1: Supplementary Tables.**

**Additional file 2: Supplementary Figure 1.** IPA comparison analysis across TBI-GT3 vs. Control and TBI-Veh vs. Control. Differentially activated pathways are represented in orange and repressed in blue. Hierarchical clustering was applied to both pathways. **Supplementary Figure 2.** IPA enrichment analysis of the genes differentially expressed in the PBI-Veh and PBI-comparison. The Y-axis gives the negative logarithm function of Benjamini-Hochberg (B-H) false discovery rate *p*-value. Orange or shades of orange bars indicate the predicted activation state of the canonical pathway, and blue or lighter shades of the blue bar indicate a negative z-score and down-regulation of the pathway. **Supplementary Figure 3.** IPA enrichment analysis of the genes differentially expressed in the PBI-GT3 and Control comparison. The Y-axis gives the negative logarithm function of Benjamini-Hochberg (B-H) false discovery rate *p*-value. Orange or shades of orange bars indicate the predicted activation state of the canonical pathway, and blue or lighter shades of the blue bar indicate a negative z-score and down-regulation of the pathway. **Supplementary Figure 4.** IPA comparison analysis across PBI-GT3 vs. Control and PBI-Veh vs. Control. Differentially activated pathways is represented in orange and repressed in blue. Hierarchical clustering was applied to both pathways. **Supplementary Figure 5.** IPA analysis of male and female NHPs administered with the vehicle and exposed to PBI. The Y-axis gives the negative logarithm function of Benjamini-Hochberg (B-H) false discovery rate *p*-value. Orange or shades of orange bars indicate the predicted activation state of the canonical pathway, and blue or lighter shades of the blue bar indicate a negative z-score and down-regulation of the pathway. **Supplementary Figure 6.** IPA comparison analysis across TBI-Vehicle vs. Control and PBI-Vehicle vs. Control. Differentially activated pathways is represented in orange and repressed in blue. Hierarchical clustering was applied to both pathways. **Supplementary Figure 7A.** Upset plot showing the overlap between Lung and jejunum tissues after TBI.** B**: IPA comparison analysis across Jejunum TBI and lung TBI. Differentially activated pathways is represented in orange and repressed in blue. Hierarchical clustering was applied to both pathways. **Supplementary Figure 8A.** Upset plot showing the overlap between Lung and jejunum tissues after PBI. B: IPA comparison analysis across Jejunum PBI and lung PBI. Differentially activated pathways is represented in orange and repressed in blue. Hierarchical clustering was applied to both pathways.

## Data Availability

All data generated or analyzed during this study are included in this published article (and its Supplementary Information files). RNAseq Fastq files are uploaded in the NCBI SRA database with BioProject accession number: PRJNA875045 (https://dataview.ncbi.nlm.nih.gov/object/PRJNA875045?reviewer=7mj8vq779cbct2oj8hirsrgr30).

## References

[1] Dorr H, Meineke V (2011). Acute radiation syndrome caused by accidental radiation exposure - therapeutic principles. BMC Med.

[2] Anno GH, Young RW, Bloom RM, Mercier JR (2003). Dose response relationships for acute ionizing-radiation lethality. Health Phys.

[3] McCann DGC (2006). Radiation poisoning: Current concepts in the acute radiation syndrome. Am J Clin Med.

[4] Armed Forces Radiobiology Research Institute: Medical management of radiological casualities, Fourth edn. Bethesda: Armed Forces Radiobiology Research Institute; 2013.

[5] Singh VK, Seed TM (2021). Radiation countermeasures for hematopoietic acute radiation syndrome: growth factors, cytokines and beyond. Int J Radiat Biol.

[6] U.S. Food and Drug Administration. Animal rule approvals. 2022. https://www.fda.gov/drugs/nda-and-bla-approvals/animal-rule-approvals.

[7] Singh VK, Beattie LA, Seed TM (2013). Vitamin E: Tocopherols and tocotrienols as potential radiation countermeasures. J Radiat Res.

[8] Singh VK, Hauer-Jensen M (2016). Gamma-tocotrienol as a promising countermeasure for acute radiation syndrome: Current status. Int J Mol Sci.

[9] Berbee M, Fu Q, Boerma M, Sree Kumar K, Loose DS, Hauer-Jensen M (2012). Mechanisms underlying the radioprotective properties of gamma-tocotrienol: comparative gene expression profiling in tocol-treated endothelial cells. Genes Nutr.

[10] Berbee M, Fu Q, Boerma M, Wang J, Kumar KS, Hauer-Jensen M (2009). Gamma-Tocotrienol ameliorates intestinal radiation injury and reduces vascular oxidative stress after total-body irradiation by an HMG-CoA reductase-dependent mechanism. Radiat Res.

[11] Singh VK, Kulkarni S, Fatanmi OO, Wise SY, Newman VL, Romaine PL, Hendrickson H, Gulani J, Ghosh SP, Kumar KS (2016). Radioprotective efficacy of gamma-tocotrienol in nonhuman primates. Radiat Res.

[12] Singh VK, Seed TM (2023). Development of gamma-tocotrienol as a radiation medical countermeasure for the acute radiation syndrome: current status and future perspectives. Expert Opin Investig Drugs.

[13] Ghosh SP, Kulkarni S, Hieber K, Toles R, Romanyukha L, Kao TC, Hauer-Jensen M, Kumar KS (2009). Gamma-tocotrienol, a tocol antioxidant as a potent radioprotector. Int J Radiat Biol.

[14] U.S. Food and Drug Administration. Animal rule information. 2023. http://www.fda.gov/EmergencyPreparedness/Counterterrorism/MedicalCountermeasures/MCMRegulatoryScience/ucm391604.htm.

[15] Singh VK, Olabisi AO (2017). Nonhuman primates as models for the discovery and development of radiation countermeasures. Expert Opin Drug Discov.

[16] Chaudhry MA (2008). Biomarkers for human radiation exposure. J Biomed Sci.

[17] Sullivan JM, Prasanna PG, Grace MB, Wathen LK, Wallace RL, Koerner JF, Coleman CN (2013). Assessment of biodosimetry methods for a mass-casualty radiological incident: medical response and management considerations. Health Phys.

[18] Singh VK, Seed TM, Cheema AK (2021). Metabolomics-based predictive biomarkers of radiation injury and countermeasure efficacy: Current status and future perspectives. Expert Rev Mol Diagn.

[19] Singh VK, Newman VL, Romaine PL, Hauer-Jensen M, Pollard HB (2016). Use of biomarkers for assessing radiation injury and efficacy of countermeasures. Expert Rev Mol Diagn.

[20] Pannkuk EL, Fornace AJ, Laiakis EC (2017). Metabolomic applications in radiation biodosimetry: exploring radiation effects through small molecules. Int J Radiat Biol.

[21] Amundson SA. Transcriptomics for radiation biodosimetry: progress and challenges. Int J Radiat Biol. 2023;99(6):925–33. 10.1080/09553002.2021.1928784 PMC10026363 33970766

[22] Amundson SA. The transcriptomic revolution and radiation biology. Int J Radiat Biol. 2022;98(3):428–33. 10.1080/09553002.2021.1987562 PMC9520858 34586968

[23] Port M, Herodin F, Drouet M, Valente M, Majewski M, Ostheim P, Lamkowski A, Schule S, Forcheron F, Tichy A (2021). Gene expression changes in irradiated baboons: A summary and interpretation of a decade of findings. Radiat Res.

[24] Moore R, Puniya BL, Powers R, Guda C, Bayles KW, Berkowitz D, Helikar T (2021). Integrative network analyses of transcriptomics data reveal potential drug targets for acute radiation syndrome. Sci Rep.

[25] Baranov AE, Selidovkin GD, Butturini A, Gale RP (1994). Hematopoietic recovery after 10-Gy acute total body radiation. Blood.

[26] Drouet M, Herodin F (2010). Radiation victim management and the haematologist in the future: time to revisit therapeutic guidelines?. Int J Radiat Biol.

[27] Hirama T, Tanosaki S, Kandatsu S, Kuroiwa N, Kamada T, Tsuji H, Yamada S, Katoh H, Yamamoto N, Tsujii H (2003). Initial medical management of patients severely irradiated in the Tokai-mura criticality accident. Br J Radiol.

[28] Cai Y, Wang W, Liang H, Sun L, Teitelbaum DH, Yang H (2013). Keratinocyte growth factor pretreatment prevents radiation-induced intestinal damage in a mouse model. Scand J Gastroenterol.

[29] de Carvalho AD, de Souza W, Morgado-Diaz JA (2006). Morphological and molecular alterations at the junctional complex in irradiated human colon adenocarcinoma cells, Caco-2. Int J Radiat Biol.

[30] Dublineau I, Lebrun F, Grison S, Griffiths NM (2004). Functional and structural alterations of epithelial barrier properties of rat ileum following X-irradiation. Can J Physiol Pharmacol.

[31] Lee B, Moon KM, Kim CY (2018). Tight junction in the intestinal epithelium: Its association with diseases and regulation by phytochemicals. J Immunol Res.

[32] Gruber S, Cini N, Kowald LM, Mayer J, Rohorzka A, Kuess P, Dorr W (2018). Upregulated epithelial junction expression represents a novel parameter of the epithelial radiation response to fractionated irradiation in oral mucosa. Strahlenther Onkol.

[33] Yu FX, Guan KL (2013). The Hippo pathway: regulators and regulations. Genes Dev.

[34] Gregorieff A, Liu Y, Inanlou MR, Khomchuk Y, Wrana JL (2015). Yap-dependent reprogramming of Lgr5(+) stem cells drives intestinal regeneration and cancer. Nature.

[35] Peter RU, Beetz A, Ried C, Michel G, van Beuningen D, Ruzicka T (1993). Increased expression of the epidermal growth factor receptor in human epidermal keratinocytes after exposure to ionizing radiation. Radiat Res.

[36] Sheridan MT, O’Dwyer T, Seymour CB, Mothersill CE. Potential indicators of radiosensitivity in squamous cell carcinoma of the head and neck. Radiat Oncol Investig. 1997;5(4):180–6. 10.1002/(SICI)1520-6823(1997)5:4<180::AID-ROI3>3.0.CO;2-U 9327497

[37] Lammering G, Valerie K, Lin PS, Mikkelsen RB, Contessa JN, Feden JP, Farnsworth J, Dent P, Schmidt-Ullrich RK (2001). Radiosensitization of malignant glioma cells through overexpression of dominant-negative epidermal growth factor receptor. Clin Cancer Res.

[38] Milas L, Fan Z, Andratschke NH, Ang KK (2004). Epidermal growth factor receptor and tumor response to radiation: in vivo preclinical studies. Int J Radiat Oncol Biol Phys.

[39] Liu YG, Chen JK, Zhang ZT, Ma XJ, Chen YC, Du XM, Liu H, Zong Y, Lu GC (2017). NLRP3 inflammasome activation mediates radiation-induced pyroptosis in bone marrow-derived macrophages. Cell Death Dis.

[40] Smith AO, Ju W, Adzraku SY, Wenyi L, Yuting C, Qiao J, Xu K, Zeng L (2021). Gamma radiation induce inflammasome signaling and pyroptosis in microvascular endothelial cells. J Inflamm Res.

[41] Bell BI, Vercellino J, Brodin NP, Velten C, Nanduri LSY, Tanaka KE, Fang Y, Wang Y, Macedo R, English J (2022). Increased relative biological effectiveness of orthovoltage X-rays compared to γ-rays in preclinical Irradiation. Can Res.

[42] Vellichirammal NN, Sethi S, Pandey S, Singh J, Wise SY, Carpenter AD, Fatanmi OO, Guda C, Singh VK: Lung transcriptome of nonhuman primates exposed to total- and partial-body irradiation. Mol Ther Nucleic Acids. 2021; 29. (In Press). 10.1016/j.omtn.2022.08.006 PMC9418744 36090752

[43] Beach T, Authier S, Javitz HS, Wong K, Bakke J, Gahagen J, Bunin DI, Chang PY (2021). Total body irradiation models in NHPs - consideration of animal sex and provision of supportive care to advance model development. Int J Radiat Biol.

[44] Singh VK, Carpenter AD, Janocha BL, Petrus SA, Fatanmi OO, Wise SY, Seed TM. Radiosensitivity of rhesus nonhuman primates: Consideration of sex, supportive care, body weight and age at time of exposure. Expert Opin Drug Discov. 2023. in press. 10.1080/17460441.2023.2205123. 10.1080/17460441.2023.2205123 PMC10330264 37073409

[45] Cheema AK, Mehta KY, Santiago PT, Fatanmi OO, Kaytor MD, Singh VK (2019). Pharmacokinetic and metabolomic studies with BIO 300, a nanosuspension of genistein, in a nonhuman primate model. Int J Mol Sci.

[46] Li Y, Singh J, Varghese R, Zhang Y, Fatanmi OO, Cheema AK, Singh VK (2021). Transcriptome of rhesus macaque (Macaca mulatta) exposed to total-body irradiation. Sci Rep.

[47] National Research Council of the National Academy of Sciences: Guide for the care and use of laboratory animals, 8th edn. Washington, DC: National Academies Press; 2011.

[48] Li Y, Girgis M, Wise SY, Fatanmi OO, Seed TM, Maniar M, Cheema AK, Singh VK (2021). Analysis of the metabolomic profile in serum of irradiated nonhuman primates treated with Ex-Rad, a radiation countermeasure. Sci Rep.

[49] Garg S, Garg TK, Wise SY, Fatanmi OO, Miousse IR, Savenka AV, Basnakian AG, Singh VK, Hauer-Jensen M. Effects of gamma-tocotrienol on intestinal injury in a GI-specific acute radiation syndrome model in nonhuman primate. Int J Mol Sci. 2022;23(9):4643. 10.3390/ijms23094643 PMC9100017 35563033

[50] American Veterinary Medical Association. AVMA guidelines for the euthanasia of animals: 2020 edition. 2020. https://www.avma.org/sites/default/files/2020-01/2020-Euthanasia-Final-1-17-20.pdf.

[51] Babraham Institute. FastQC: A quality control tool for high throughput sequence data. 2019. https://www.bioinformatics.babraham.ac.uk/projects/fastqc/.

[52] Dobin A, Davis CA, Schlesinger F, Drenkow J, Zaleski C, Jha S, Batut P, Chaisson M, Gingeras TR (2013). STAR: ultrafast universal RNA-seq aligner. Bioinformatics.

[53] Love MI, Huber W, Anders S (2014). Moderated estimation of fold change and dispersion for RNA-seq data with DESeq2. Genome Biol.

[54] Kramer A, Green J, Pollard J, Tugendreich S (2014). Causal analysis approaches in Ingenuity Pathway Analysis. Bioinformatics.

[55] Ge SX, Jung D, Yao R (2020). ShinyGO: a graphical gene-set enrichment tool for animals and plants. Bioinformatics.

