# Supplementary material for: Transcriptome profile changes in the jejunum of nonhuman primates exposed to supralethal dose of total- or partial-body radiation
Source: BMC Genomics. 2023 May 22;24:274. doi: 10.1186/s12864-023-09385-3 (PMC10201771; doi:10.1186/s12864-023-09385-3)
Supplement: Supplementary file 2 — Additional file 2: Supplementary Figure 1. IPA comparison analysis across TBI-GT3 vs. Control and TBI-Veh vs. Control. Differentially activated pathways are represented in orange and repressed in blue. Hierarchical clustering was applied to both pathways. Supplementary Figure 2. IPA enrichment analysis of the genes differentially expressed in the PBI-Veh and PBI-comparison. The Y-axis gives the negative logarithm function of Benjamini-Hochberg (B-H) false discovery rate p-value. Orange or shades of orange bars indicate the predicted activation state of the canonical pathway, and blue or lighter shades of the blue bar indicate a negative z-score and down-regulation of the pathway. Supplementary Figure 3. IPA enrichment analysis of the genes differentially expressed in the PBI-GT3 and Control comparison. The Y-axis gives the negative logarithm function of Benjamini-Hochberg (B-H) false discovery rate p-value. Orange or shades of orange bars indicate the predicted activation state of the canonical pathway, and blue or lighter shades of the blue bar indicate a negative z-score and down-regulation of the pathway. Supplementary Figure 4. IPA comparison analysis across PBI-GT3 vs. Control and PBI-Veh vs. Control. Differentially activated pathways is represented in orange and repressed in blue. Hierarchical clustering was applied to both pathways. Supplementary Figure 5. IPA analysis of male and female NHPs administered with the vehicle and exposed to PBI. The Y-axis gives the negative logarithm function of Benjamini-Hochberg (B-H) false discovery rate p-value. Orange or shades of orange bars indicate the predicted activation state of the canonical pathway, and blue or lighter shades of the blue bar indicate a negative z-score and down-regulation of the pathway. Supplementary Figure 6. IPA comparison analysis across TBI-Vehicle vs. Control and PBI-Vehicle vs. Control. Differentially activated pathways is represented in orange and repressed in blue. Hierarchical clustering w [file 12864_2023_9385_MOESM2_ESM.pdf]

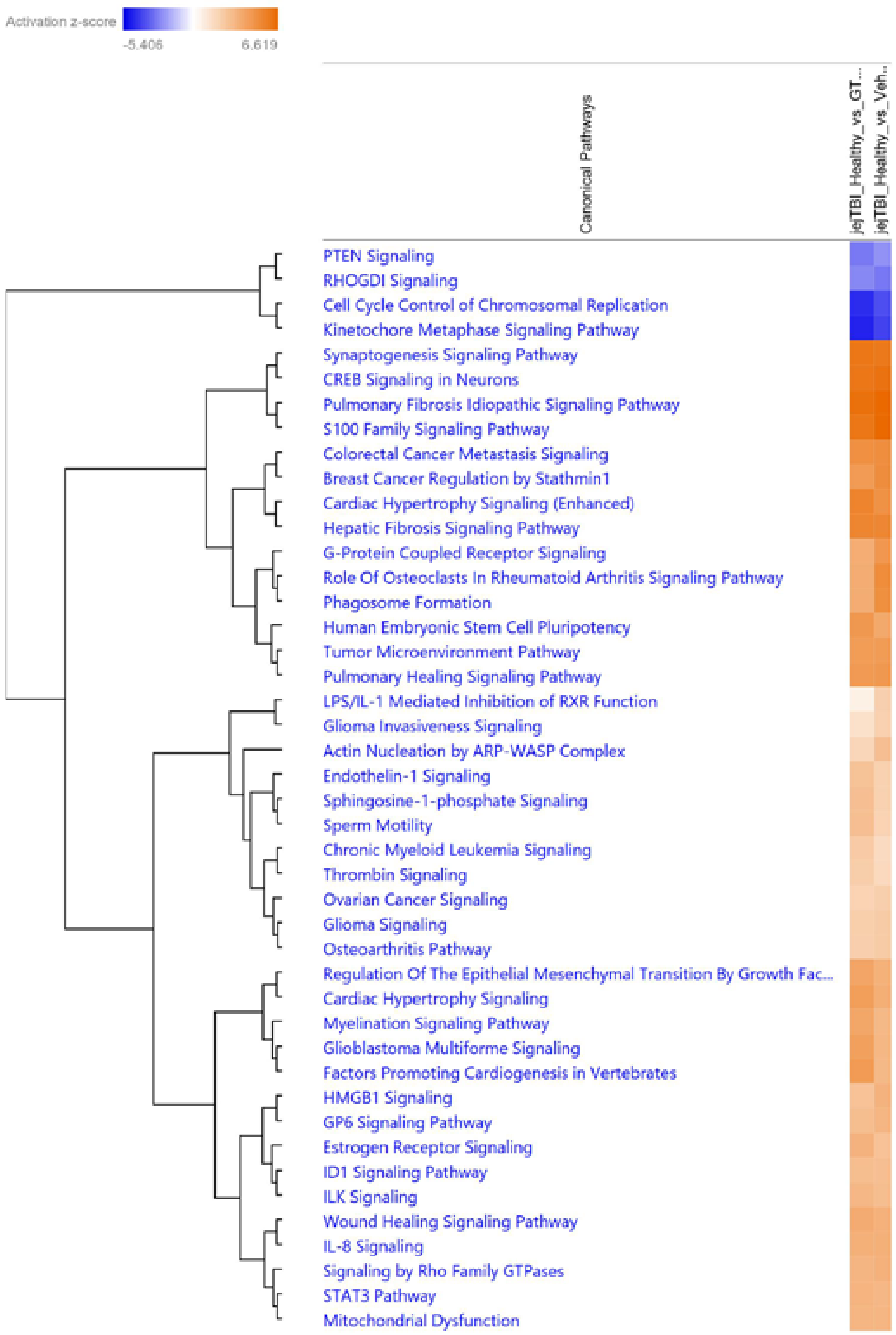

Supplementary Figure 1: IPA comparison analysis across TBI-GT3 vs. Control and TBI-Veh vs. Control. Differentially activated pathways is represented in orange and repressed in blue. Hierarchical clustering was applied to both pathways

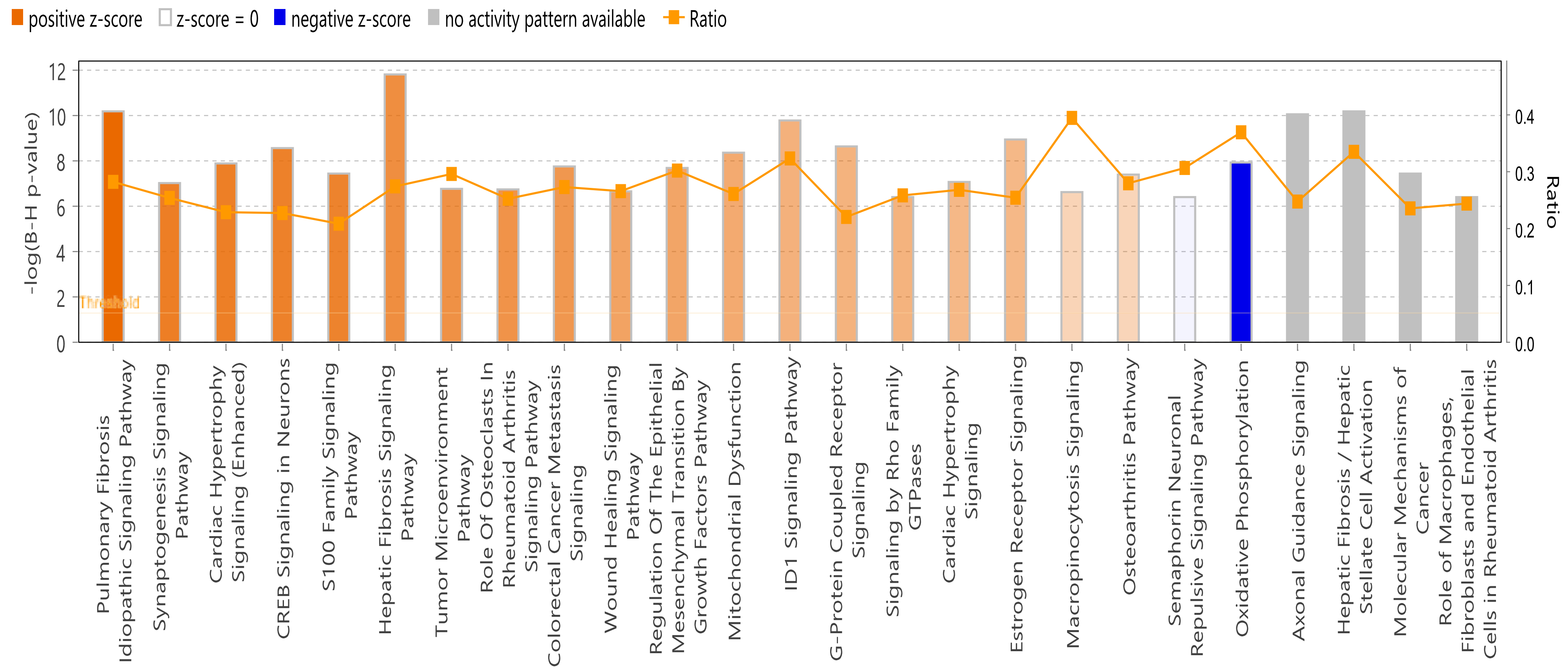

© 2000-2023 QIAGEN. All rights reserved.

Supplementary Figure 2: IPA enrichment analysis of the genes differentially expressed in the PBI-Veh and PBI-comparison. The Y-axis gives the negative logarithm function of Benjamini-Hochberg (B-H) false discovery rate p-value. Orange or shades of orange bars indicate the predicted activation state of the canonical pathway, and blue or lighter shades of the blue bar indicate a negative z-score and down-regulation of the pathway.

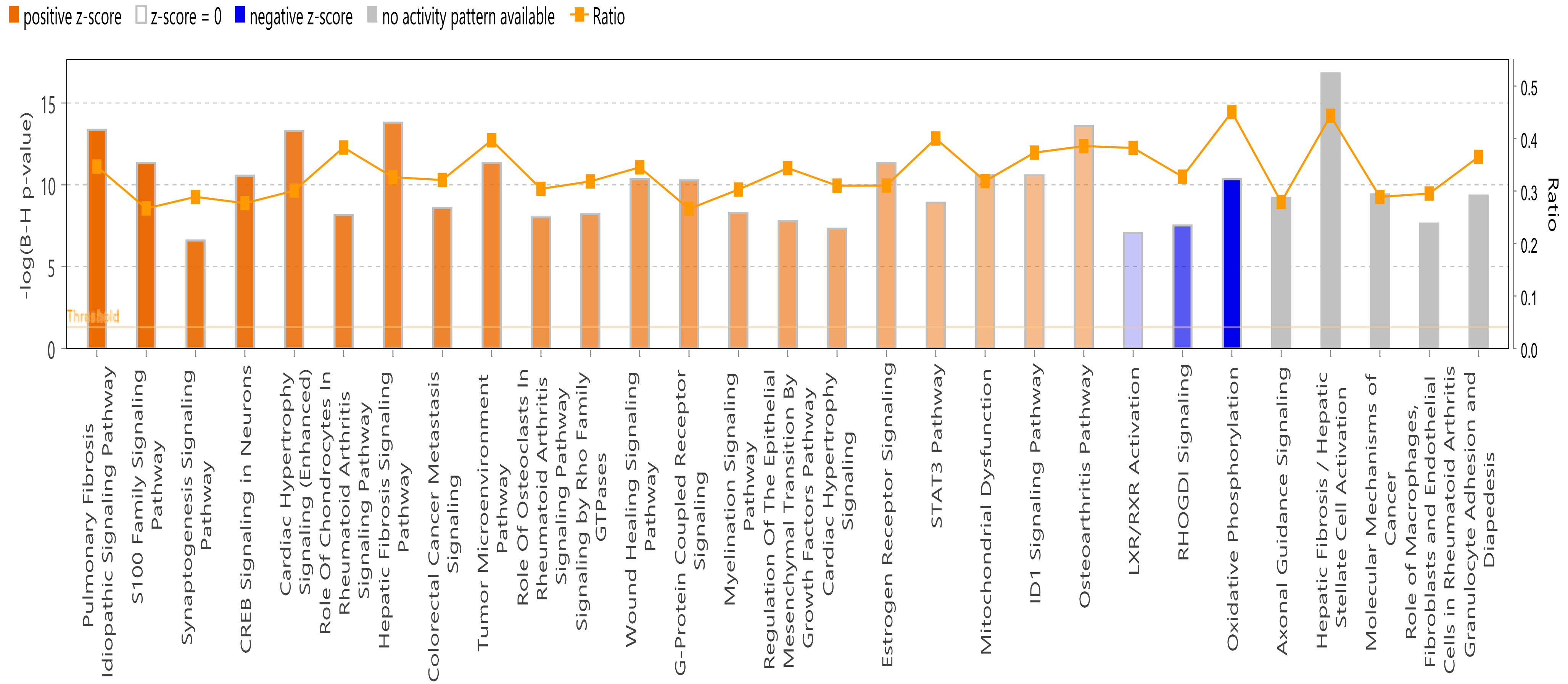

© 2000-2023 QIAGEN. All rights reserved.

Supplementary Figure 3: IPA enrichment analysis of the genes di erentially expressed in the PBI-GT3 and Control comparison. The Y-axis gives the negative logarithm function of Benjamini-Hochberg (B-H) false discovery rate p-value. Orange or shades of orange bars indicate the predicted activation state of the canonical pathway, and blue or lighter shades of the blue bar indicate a negative z-score and down-regulation of the pathway.

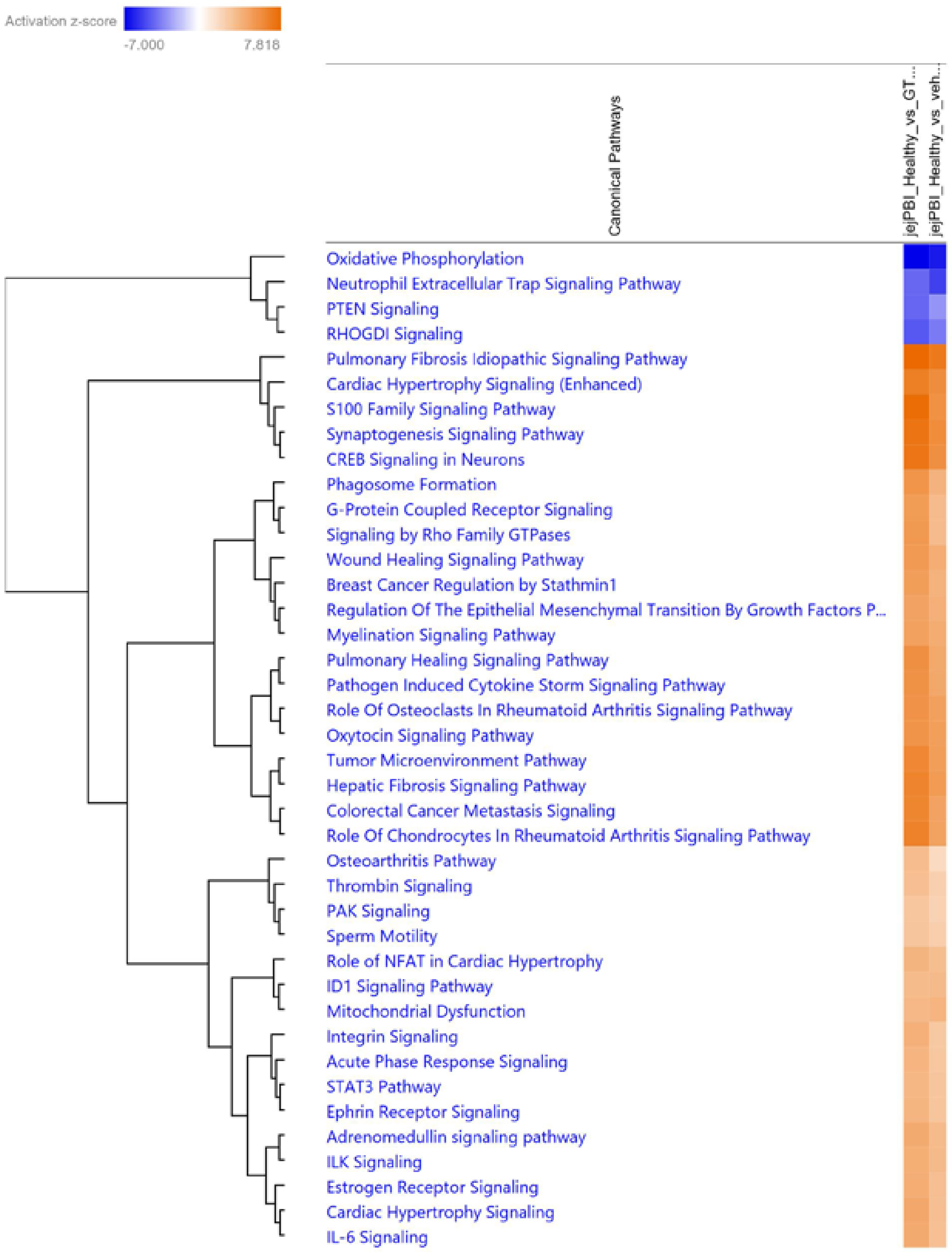

Supplementary Figure 4: IPA comparison analysis across PBI-GT3 vs. Control and PBI-Veh vs. Control. Differentially activated pathways is represented in orange and repressed in blue. Hierarchical clustering was applied to both pathways

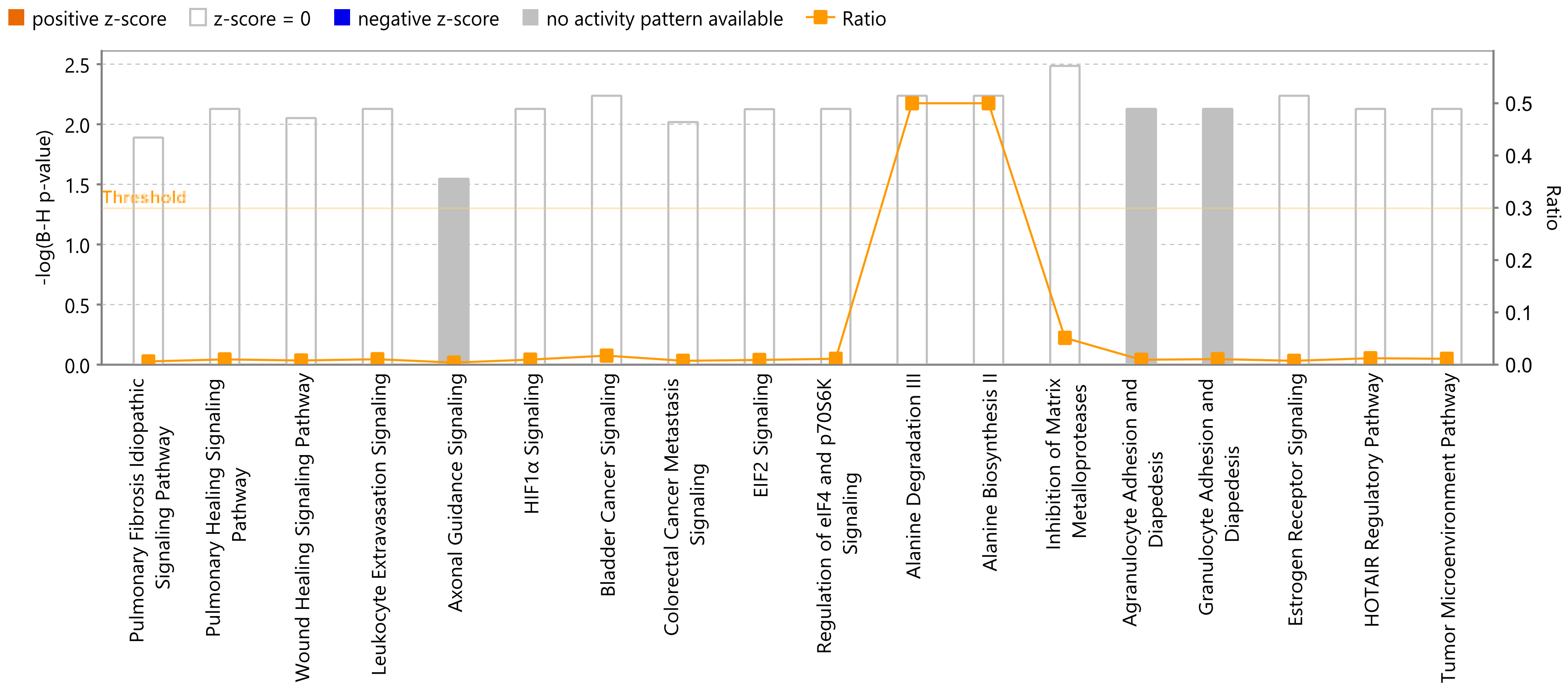

© 2000-2022 QIAGEN. All rights reserved.

ISupplementary Figure 5: IPA analysis of male and female NHPs administered with the vehicle and exposed to PBI. The Y-axis gives the negative logarithm function of Benjamini-Hochberg (B-H) false discovery rate p-value. Orange or shades of orange bars indicate the predicted activation state of the canonical pathway, and blue or lighter shades of the blue bar indicate a negative z-score and down-regulation of the pathway.

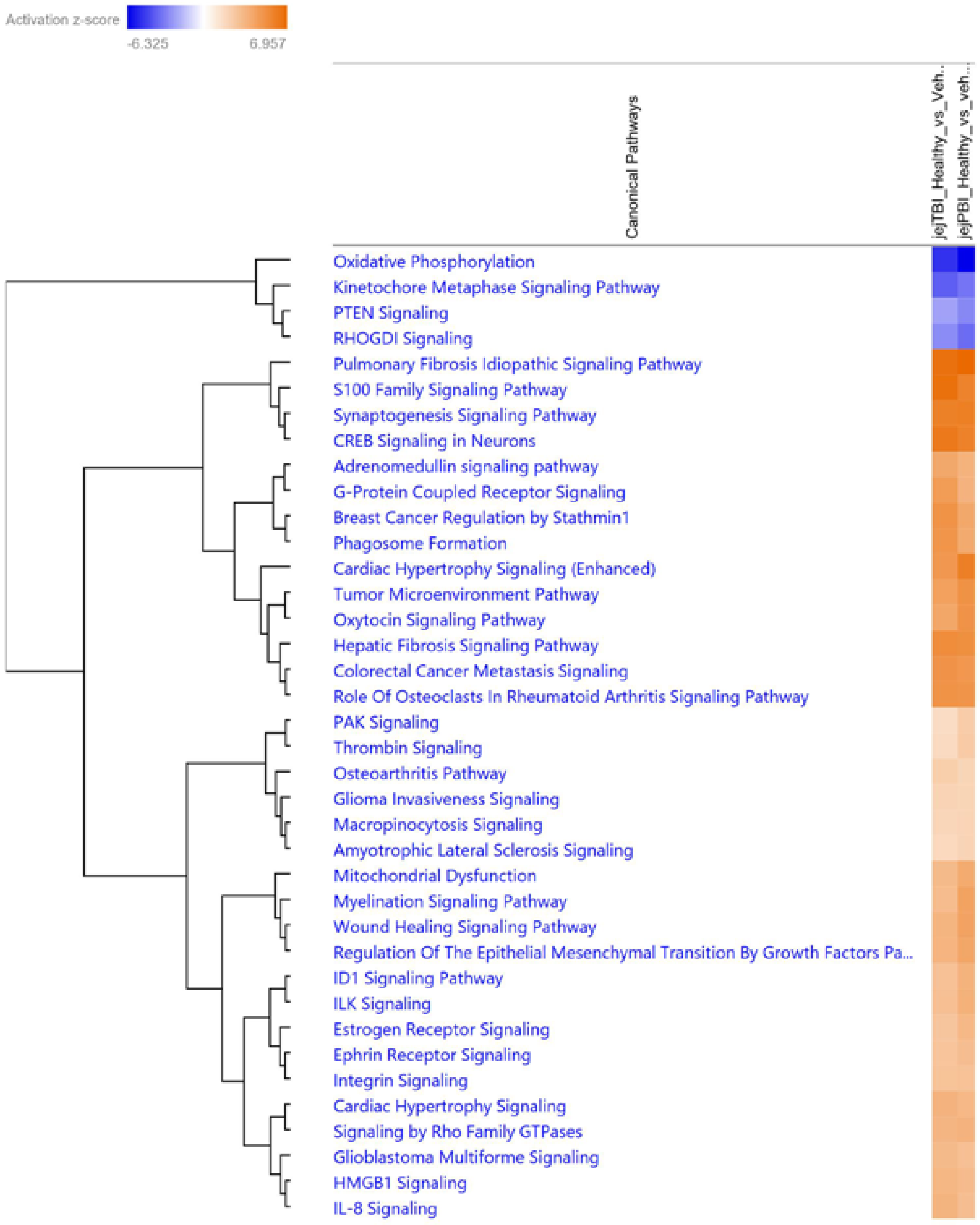

Supplementary Figure 6: IPA comparison analysis across TBI-Vehicle vs. Control and PBI-Vehicle vs. Control. Differentially activated pathways is represented in orange and repressed in blue. Hierarchical clustering was applied to both pathways.

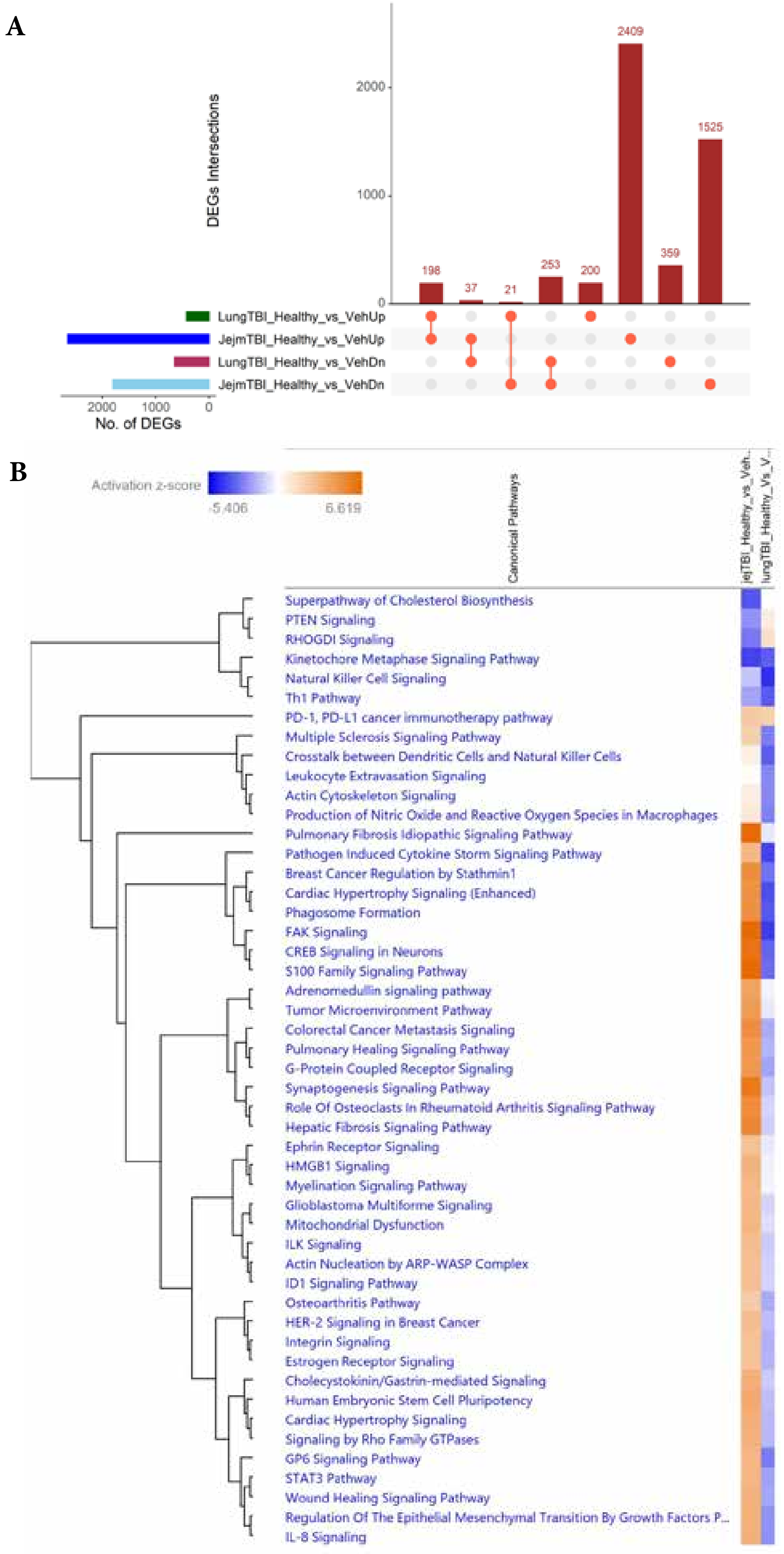

Supplementary Figure 7A: Upset plot showing the overlap between Lung and jejunum tissues after TBI. B: IPA comparison analysis across Jejunum TBI and lung TBI. Differentially activated pathways is represented in orange and repressed in blue. Hierarchical clustering was applied to both pathways

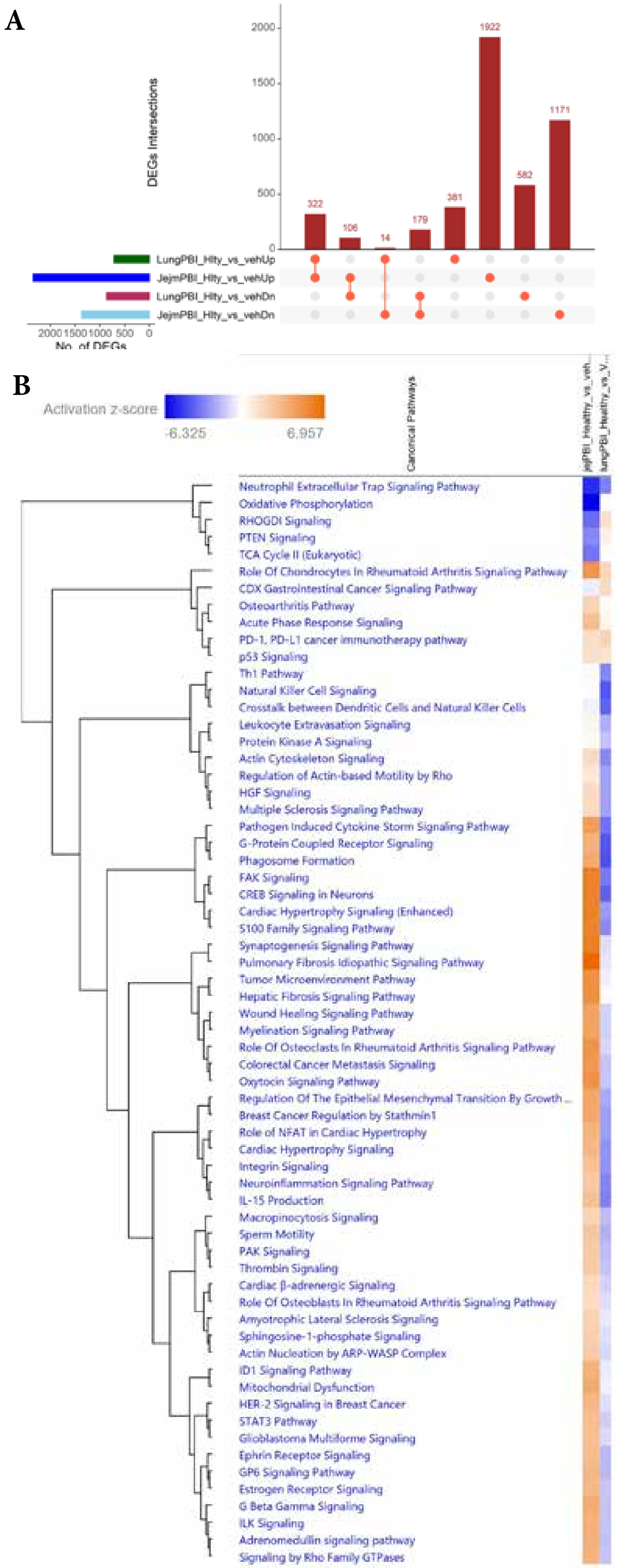

Supplementary Figure 8A: Upset plot showing the overlap between Lung and jejunum tissues after PBI. B: IPA comparison analysis across Jejunum PBI and lung PBI. Differentially activated pathways is represented in orange and repressed in blue. Hierarchical clustering was applied to both pathways
